# Real-Time Dynamics of Emerging Actin Networks in Cell-Mimicking Compartments

**DOI:** 10.1371/journal.pone.0116521

**Published:** 2015-03-18

**Authors:** Siddharth Deshpande, Thomas Pfohl

**Affiliations:** Department of Chemistry, University of Basel, Klingelbergstrasse 80, CH-4056 Basel, Switzerland; Dalhousie University, CANADA

## Abstract

Understanding the cytoskeletal functionality and its relation to other cellular components and properties is a prominent question in biophysics. The dynamics of actin cytoskeleton and its polymorphic nature are indispensable for the proper functioning of living cells. Actin bundles are involved in cell motility, environmental exploration, intracellular transport and mechanical stability. Though the viscoelastic properties of actin-based structures have been extensively probed, the underlying microstructure dynamics, especially their disassembly, is not fully understood. In this article, we explore the rich dynamics and emergent properties exhibited by actin bundles within flow-free confinements using a microfluidic set-up and epifluorescence microscopy. After forming entangled actin filaments within cell-sized quasi two-dimensional confinements, we induce their bundling using three different fundamental mechanisms: counterion condensation, depletion interactions and specific protein-protein interactions. Intriguingly, long actin filaments form emerging networks of actin bundles via percolation leading to remarkable properties such as stress generation and spindle-like intermediate structures. Simultaneous sharing of filaments in different links of the network is an important parameter, as short filaments do not form networks but segregated clusters of bundles instead. We encounter a hierarchical process of bundling and its subsequent disassembly. Additionally, our study suggests that such percolated networks are likely to exist within living cells in a dynamic fashion. These observations render a perspective about differential cytoskeletal responses towards numerous stimuli.

## Introduction

The mechanical integrity of a cell is orchestrated by the cytoskeleton, a dynamic system mainly comprised of filamentous actin (F-actin), microtubules and intermediate filaments, aided by a plethora of accessory proteins. Actin, a dynamic and polymorphic component, forms a variety of structures such as filaments, bundles and their networks [1]. Bundling is a process in which two or more actin filaments join together along their longitudinal axes to form a thicker and more rigid rod-like structure known as a ‘bundle’. Actin bundles are found in specialized structures such as filopodia, bristles, microvilli, stereocilia [2] and in the growth cones of axons and dendrites [3]; furthermore, they are abundant in plant cells [4]. The unique viscoelastic properties shown by actin-based structures have been extensively probed by various means. This has yielded some major insights into their fundamental properties, such as the viscoelastic behavior of cross-linked and bundled networks using macrorheology [5–7], differential mechanical response of actin bundles to forces using optical tweezers [8], the control of bundle stiffness by actin binding proteins (ABPs) [9], and the effect of cyclic shear on the networks [10]. Yet, the underlying microstructure dynamics that are responsible for these mechanical properties have received less attention, especially the disassembly of actin networks [11]. With this background, we aimed at studying in real time the evolution of actin microstructures in confined volumes in presence of various bundling agents.

To study reversible reaction sequences (assembly and disassembly) in a step-by-step manner, one needs an open system. As a result, there have been relatively few studies in this direction, as most of the experimental systems are closed, such as sealed coverslips [6, 12], hermetically sealed chambers [13], emulsion droplets [7] and liposomes [14]. Though actin filament dynamics in flow and confinements have been investigated [15–19], the collective behavior of confined actin filaments in a stepwise manner is largely unexplored, which we aimed to address.

Here, we present the dynamics of emerging networks of actin bundles using time-lapse epifluorescence microscopy with high spatiotemporal resolution. We studied actin networks formed by three different actin bundling agents known to have distinct bundling mechanisms: Mg^2+^ ions causing counterion condensation; polyethylene glycol (PEG) polymers leading to depletion interactions; and filamin dimers specifically interacting with F-actin. We find a common thread in the form of a hierarchical process where filaments fuse together to form ‘small bundles’ which further coalesce to form ‘bigger bundles’, during network formation. The evolution of reaction components guides us to the reaction kinetics and the involved mechanisms. Based on the data, we have built kinetic models to explain the observed actin dynamics. We have also performed local analyses to gain insights into intriguing phenomena, such as actin filament zipping, stress generation and spindle-like structure formation, observed during the network formation within confined environments.

## Results

**Fig 1:**
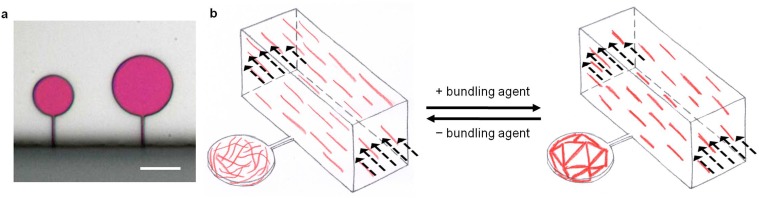
The microfluidic set-up and the schematic experimental plan. (a) Zoomed-in view of a silicon wafer master showing a circular microchamber. The quasi two-dimensional (*h* = 0.5 μm) microchamber is attached to the controlling channel (*h* = 5.5 μm) via a narrow connecting channel (0.5 μm wide). The scale bar represents 10 μm. (b) In a step-by-step reaction sequence, the addition of bundling agents to a steady-state of actin filaments induces the bundling reaction resulting in a network of actin bundles while their subsequent depletion brings back the system to the original state of entangled filaments, in a diffusion-controlled manner.

In order to study the actin network dynamics, we created a straightforward microfluidic system, consisting of quasi two-dimensional (*h* = 0.5 μm), cell-sized circular compartments (diameter *d* = 5–30 μm), enclosing sub-picolitre volumes [20]. These ‘microchambers’ were connected to the controlling channel (the reservoir) via narrow connecting channels (0.5 μm wide), enabling exclusive diffusive transport into and out of the microchambers (Fig. 1a). Such a flow-free environment was ideal to form an entangled network of actin filaments in a steady-state, allowing further manipulation in a stepwise manner (Fig. 1b, Materials and Methods). For all experiments, a semi-dilute (3 μM) actin solution was used, where the actin filaments overlapped and sterically hindered each other [21]. The mesh size or the average distance between two filaments of these entangled networks of actin filaments, which depends on the actin concentration, corresponded to about 0.9 μm [7, 22]. The diffusion times of actin monomers and the bundling agents were low enough to allow homogenous distribution within the microchambers without affecting the bundling reaction rates (S1 Fig. and S1 Text).

### Actin filament length dictates emerging actin networks

**Fig 2:**
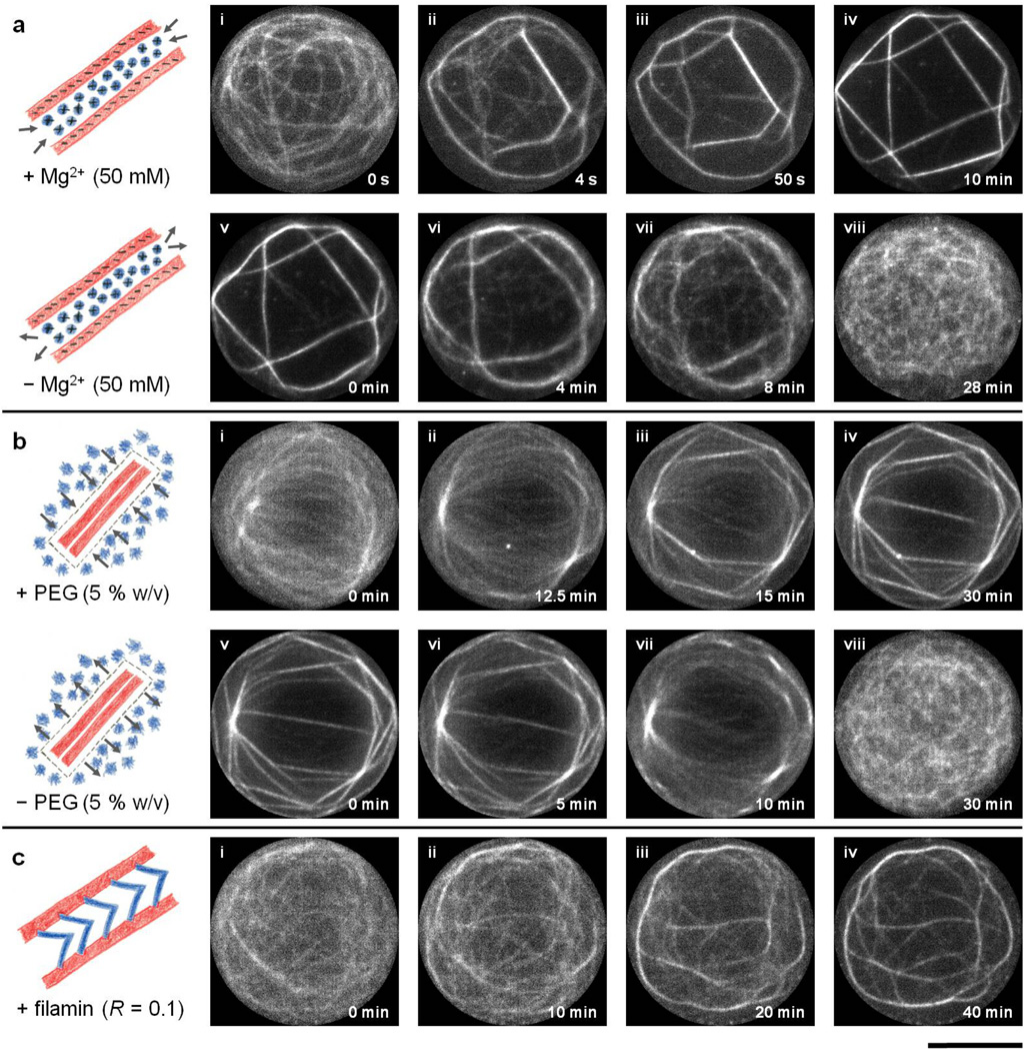
Evolution of emerging actin networks induced by counterion condensation, depletion interactions and specific binding interactions. Time-lapse images of emerging networks of actin bundles from semi-dilute solutions of confined actin filaments (3 μM, *l*_*avg*_ ≥ 10 μm) by (a) Mg^2+^ ions (50 mM), (b) PEG polymers (5% w/v, M. W. 8000) and (c) filamin dimers (ratio of filamin to actin (*R*) = 0.1). Images i-iv show the formation of networks while images v-viii show their disassembly upon addition and depletion of bundling agents respectively. Scale bar represents 10 μm.

Upon addition of bundling agents to the confined entangled actin filaments (*l*_*avg*_ = 56.7 μm ± 19.1 μm), a single network of actin bundles emerged instead of multiple isolated bundles (S1, S2, S3 Videos). Representative examples of the network formation after the addition of bundling agents are shown in Fig. 2. Note that in each case, the confined actin filaments condensed into a single network of bundles which freely fluctuated as a single entity. Actin bundles of various lengths and widths formed the ‘links’ of the networks, while the ‘nodes’ represented the junctions between two or more links (bundles). Nevertheless, in the smallest confinements (*d* = 5 μm), a closed actin bundle ring formed (S2 Fig.) exhibiting the simplest network, i.e., a single link without any nodes. Depletion of Mg^2+^ ions and PEG polymers from the microchambers successfully lead to the disassembly of the networks back to the entangled actin filaments (S4, S5 Videos), thus proving the diffusion-controlled mechanism of the device. Filamin-induced networks, however, did not disassemble due to highly specific protein-protein interactions between filamin and F-actin (S6 Video).

**Fig 3:**
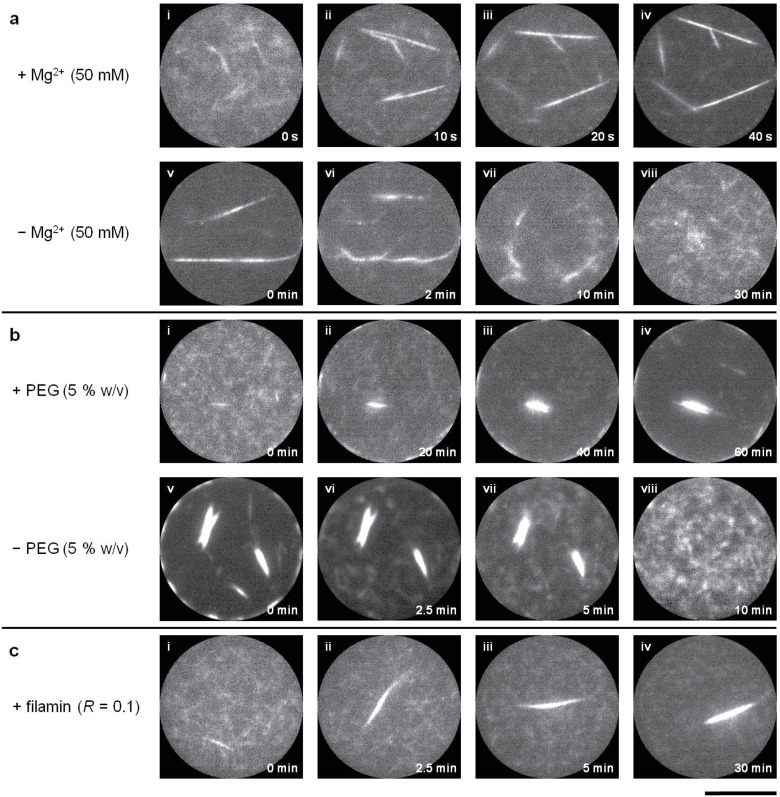
Repression of network formation by shortening the actin filament length. Time-lapse images of formation of clusters of actin bundles from semi-dilute solutions of confined actin filaments (3 μM, *l*_*avg*_ = 1 μm) by (a) Mg^2+^ ions (50 mM), (b) PEG polymers (5% w/v, M. W. 8000) and (c) filamin dimers (ratio of filamin to actin (*R*) = 0.1). Images i-iv show the formation of segregated clusters while images v-viii show their disassembly upon addition and depletion of bundling agents respectively. Scale bar represents 10 μm.

**Fig 4:**
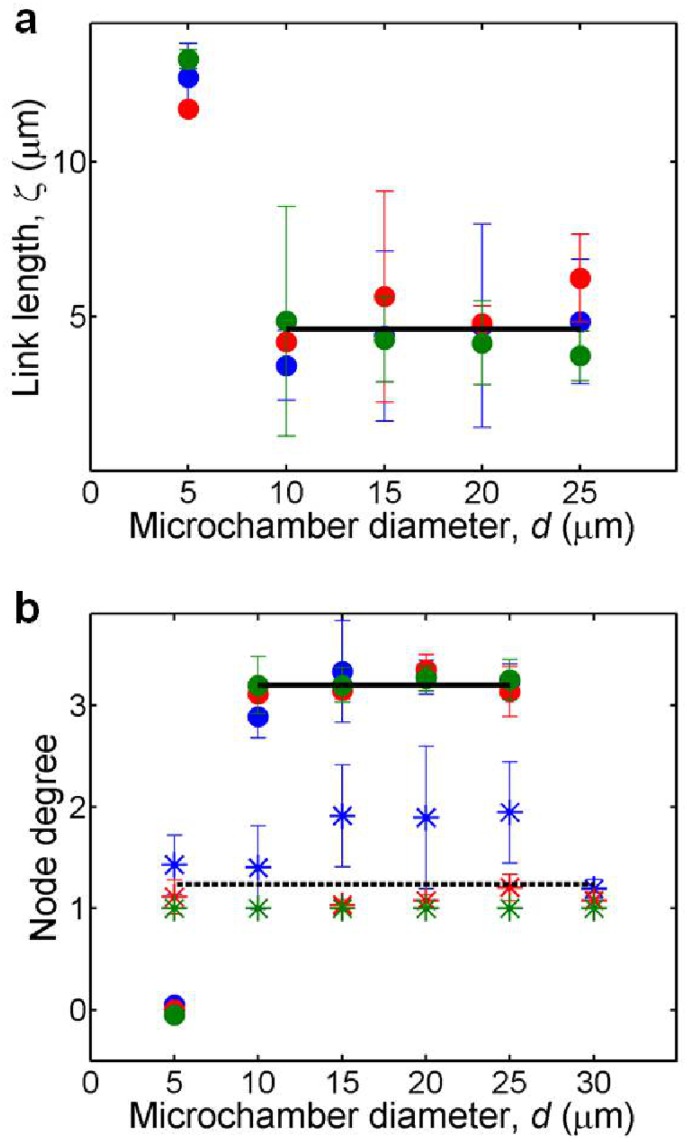
Properties of emerging networks compared with segregated clusters. (a) Average link length (*ζ*) in networks induced by Mg^2+^ ions (blue), PEG polymers (red) and filamin dimers (green). Solid line indicates the average of all three data sets for networks; the data for *d* = 5 μm is excluded. (b) Average node degree in networks (circles) and clusters (asterisks) induced by Mg^2+^ ions (blue), PEG polymers (red) and filamin dimers (green). Solid line indicates the average of all three data sets for networks; the data for *d* = 5 μm is excluded. Dashed line indicates the average of all three data sets for clusters. In total, 83 networks and 79 clusters were analyzed. Error bars indicate ± standard deviation.

Next, to test the effect of actin filament lengths on the network formation, we restricted the filament length using an appropriate concentration of gelsolin [23], an actin-severing and capping protein. There was no effect on actin network formation after restricting the length to 10 μm. However, when the length was restricted to 1 μm, it resulted in formation of isolated bundles (referred to as ‘clusters’ henceforth) as opposed to a single network (Fig. 3, S7, S8, S9 Videos). Thus, in case of short filaments, there was a dramatic repression in the network formation. As in previous experiments, depletion of the bundling agents led to the clusters disassembly (S10, S11 Videos), excluding the case of filamin-induced networks (S12 Video). In order to understand the effect of filament length on network formation/repression, we analyzed the nature of links and nodes. The average link length (*ζ*) of the emerged networks stayed almost constant (≈ 5 μm) for networks induced by each of the bundling agents, formed within different confinement sizes (Fig. 4a). The long filaments (*l*_*avg*_ > *ζ*) were easily shared between two or more links, aided by the close proximity of the entangled filaments. On the other hand, short filaments could not get shared between different links, preventing the formation of a single connected component. Thus, the filament length dictated the probability to get shared between two or more network links. The joining of different bundles to form a node was due to the simultaneous formation of bundles and the presence of shared filaments between them. Hence, sharing of filaments within different bundles was a key factor and the main cause of the emerging networks.

Thus, we found two distinct length regimes: short filaments, where a pure bundling process was observed, and long filaments where the bundling was accompanied by percolation, forming a single connected component. The two regimes could be clearly distinguished by analyzing the degree of nodes (number of incident links on a specific node) for the networks and the clusters in different confinements (Fig. 4b). Networks exhibited an average degree of about three, except in the smallest confinements showing actin bundle rings, where the degree was zero. Clusters, on the other hand, possessed an average degree of about one, suggesting that the bundles were indeed separated from each other.

### Actin networks show hierarchical assembly and disassembly

In order to elucidate the kinetics involved in the bundling and de-bundling processes, the number density of filaments inside the bundles was estimated by analyzing their fluorescence intensity profiles. A linear increase of the intensities with the number of filaments per bundle was assumed. This assumption was validated by analyzing the intensity profiles of bundles before and after fusing, where the sum of the fluorescence intensities of two smaller bundles matched up with the intensity of the bigger bundle that they ultimately formed (S3 Fig.). We found that the intensity distributions showed several discernible peaks suggesting that bundles with a specific number of filaments (3–4, 7–8, 12, 16) were more stable than others (S4 Fig.). Thus, filaments joined together to form small bundles which in turn fused to form bigger bundles, pointing towards a hierarchical bundling process. However, in case of clusters, no prominent intensity peaks were observed. To further assist the intensity profile-based analysis, we analyzed the distribution of bundles with different filament densities within the networks and clusters. Distinct small and big bundles could be clearly seen in the emerging networks, while the segregation was less clear in the case of clusters (S5 Fig.).

**Fig 5:**
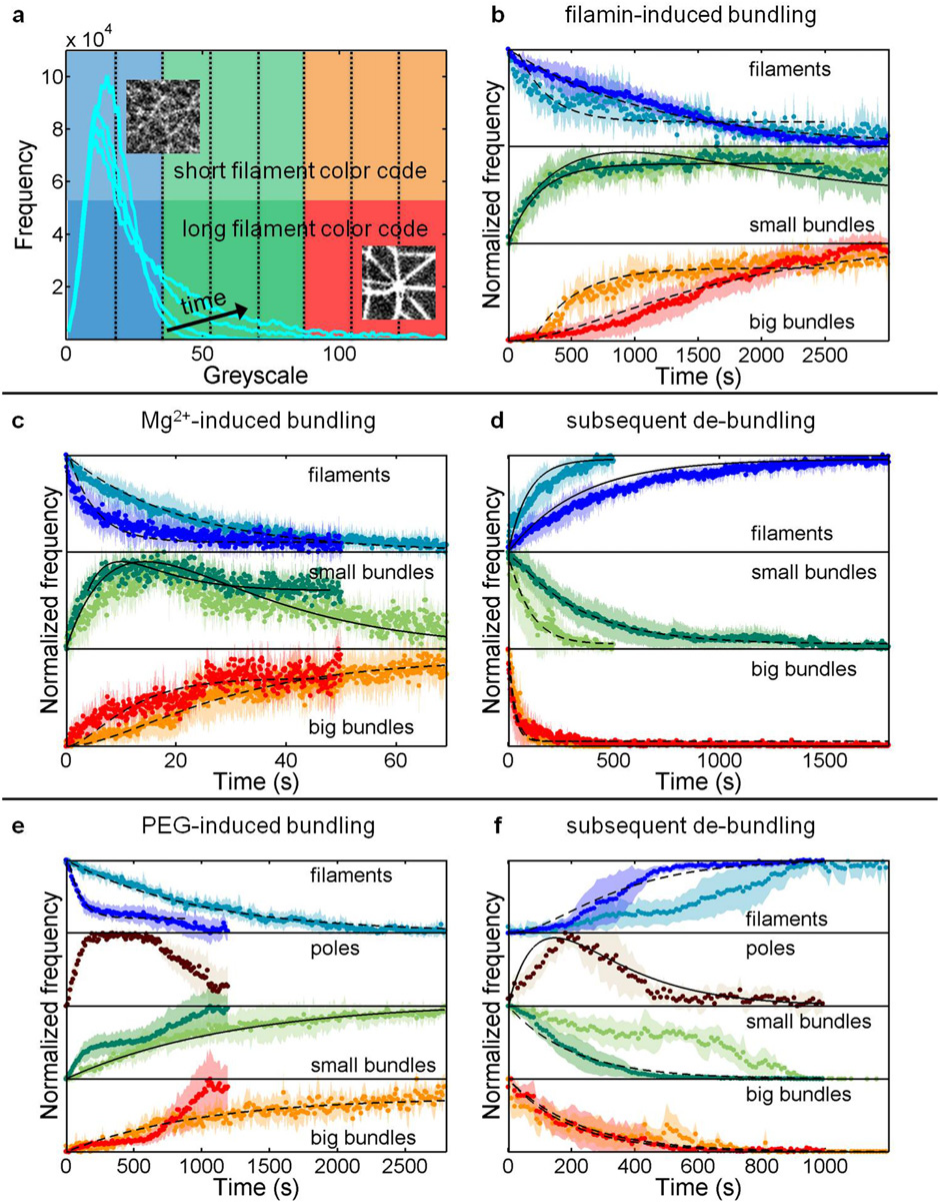
Evolution of filaments, small bundles and big bundles during the assembly and subsequent disassembly processes induced by counterion condensation, depletion interactions and specific binding interactions. (a) Typical frequency histograms (cyan) of greyscale values of the fluorescence images during the bundling process. The greyscale is divided into eight equal slices (indicated by vertical dotted lines) from which the evolution of the reaction components (b-f) is obtained. Similar scenario is applied to the de-bundling processes. (b, c, e) Evolution of reaction components during bundling induced by filamin (*n* = 12), Mg^2+^ ions (*n* = 21) and PEG polymers (*n* = 13), respectively. (d, f) Evolution of reaction components during de-bundling of networks (and clusters) induced by Mg^2+^ ions (*n* = 14) and PEG polymers (*n* = 13), respectively. The darker shades in each sub-graph correspond to network reactions while the lighter shades correspond to cluster reactions. The shaded areas indicate the errors (± standard deviation). The dotted lines are fits to the data sets as described in S2 Text. Solid lines are subsequently derived from the obtained rate constants.

To further understand the assembly dynamics, we studied the concentration changes of filaments and bundles over time, by plotting and analyzing the frequencies of greyscale values, corresponding to the fluorescence intensities. Fluorescence images of bundles showed increased occurrence of high greyscale values compared to single actin filaments (insets in Fig. 5a). Fig. 5a displays several frequency histograms, where each histogram is a time point in the bundling process. As the bundling proceeds, high greyscale values corresponding to bundles are attained.

With the aim to find possible ranges of greyscale values that represented a particular element, i.e., filaments or bundles, we divided the entire greyscale range into eight equal slices and summed up the frequencies within each slice, for each individual bundling and de-bundling event (the dotted lines in Fig. 5a). In this fashion, we detected appropriate sets of greyscale values representing specific reaction components. Fig. 5b-f display the evolution of filaments (*f*), small bundles (*b*) and big bundles (*bb*) for the three bundling mechanisms during the formation of networks (and clusters) and their disassembly back into filaments. It should be noted that the time point zero in each aforementioned graph coincides with the onset of bundling or de-bundling in the microchambers. Reactions analyzed in confinements of different diameters (*d* = 5–30 μm) are combined, since confinement size does not have a significant effect on the reaction dynamics. Though the hierarchical nature of bundling was prominent in case of networks, clusters were subjected to the same analysis for comparison. Based on the data and the step-by-step nature of assembly and disassembly $\left(f\overset{k_{1}}{\to }b\overset{k_{2}}{\to }bb\overset{k_{3}}{\to }b\overset{k_{4}}{\to }f\right)$ we built kinetic models (*k*_1_, *k*_2_, *k*_3_, *k*_4_ being the corresponding rate constants), approximating reactions to be of first order (fits shown by dashed lines in Fig. 5). Using the obtained rate constants (Table 1), the observed behavior of the reaction components could be satisfactorily explained (solid lines in Fig. 5). The kinetic models for networks and clusters by different bundling agents are described in detail in S2 Text.

**Table 1:** Rate constants obtained for bundling and de-bundling reactions.

Bundling agent	*k*_1_ (s^-1^)	*k*_2_ (s^-1^)	*k*_3_ (s^-1^)	*k*_4_ (s^-1^)
Networks				
Mg^2+^	0.21	0.13	0.032	0.003
PEG	0.01	not obtained	0.005	0.007
filamin	0.001	0.002	0	0
Clusters				
Mg^2+^	0.05	0.1	0.04	0.01
PEG	0.001	0.0008	not obtained	not obtained
filamin	0.0042	0.0023	0	0

See S2 Text for more information.

### Counterion condensation-induced emerging networks show actin filaments zipping and stressed networks

**Fig 6:**
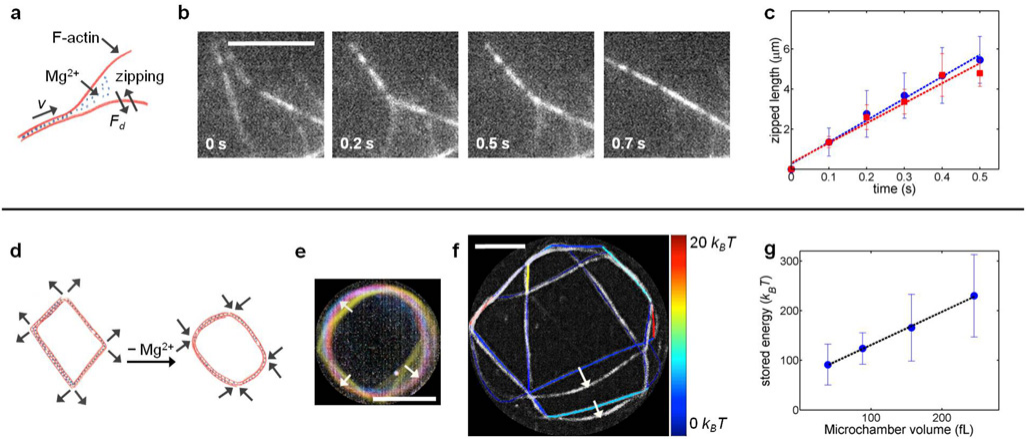
F-actin zipping leading to Mg^2+^-induced stressed actin networks. (a) Sketch of the zipping process showing that actin filaments have to overcome the drag force. (b) Time-lapse images of two single actin filaments zipping together to form an actin bundle. (c) Plot of zipped length against time. (blue circles: zipping of filaments (*n* = 14), red squares: zipping of small bundles (*n* = 8)). Error bars indicate ± standard deviation. The dashed lines are linear fits to the data. (d) Sketch depicting the energy released during the disassembly of networks subsequently used in buckling of bundles. (e) Network relaxation color-coded in time (from yellow to blue) showing the buckling of bundles (indicated by arrows) during de-bundling. (f) Network under tension (color-coded for the minimum energy stored in each of the links) overlapped on the relaxed network (in greyscale). Arrows indicate some prominent buckling of links. (g) The minimum energy stored in the networks formed in different confinement volumes (*n* = 9). Error bars indicate ± standard deviation. The dashed line shows a linear fit. Scale bars represent 5 μm.

According to Manning counterion condensation theory, the charge density on a polyelectrolyte can be neutralized by counterions in its immediate environment, since the counterions condense around the polyelectrolytes in a thin layer [24]. Actin is overall negatively charged and has a linear charge spacing of 0.25 nm [25]. Therefore, we used Mg^2+^ ions to induce actin bundling. Mg^2+^-induced bundling of long filaments proceeded quickly with most of the links already forming in the first 10 s (S1 Video). Once two filaments or two small bundles came in contact with each other, they zipped rapidly along their entire length, resulting in an accelerated bundling process, as sketched in Fig. 6a and shown as time-lapse images in Fig. 6b. This distinct accelerated bundling process was absent in the case of cluster formation, resulting in an overall slower bundling process (Table 1).

By calculating the average zipping velocity *v* of two joining filaments, the drag force *F*_*d*_ acting on each of the filaments during the bundling process was calculated according to the relation *F*_*d*_ = *γv*. Here *γ* is the drag coefficient of a cylinder in the direction perpendicular to its long axis and is defined as *γ =* 4*πηl*_*c*_/ln(2*h/r*), where *η* is the viscosity of the buffer, *l*_*c*_ is the contour length of the filament, *h* = 0.25 μm is the average height of the filament above the surface, and *r* the filament radius [26]. As the filaments overcame the drag force during bundling, it directly gave us the minimum force generated during bundling. The zipping velocities were similar for zipping of single filaments and zipping of small bundles (2–3 filaments), as shown in Fig. 6c. The average values of zipping forces obtained between two filaments and between two small bundles were thus (0.15 ± 0.03) pN and (0.16 ± 0.03) pN respectively. Our values match closely with a reported value of (0.18 ± 0.06) pN for bundling of two filaments, measured using holographic optical tweezers [27].

Sharing of long filaments between bundles (links) created tension at the nodes as the shared filaments were stretched, suppressing their thermal fluctuations. Additionally, they were acutely bent at the nodes. Thus, as the networks formed, energy was stored inside them, resulting in self-generated stress. During de-bundling, Mg^2+^ ions began to diffuse out of the network, decreasing the bending rigidity of the bundles. Eventually, the release of tension from the nodes generated enough force to buckle the now more loosely bound bundles. This tension release was seen very clearly at the start of the de-bundling process, where the whole network suddenly relaxed and the links (bundles) buckled (Fig. 2a-v). The process is sketched in Fig. 6d and color-coded in time (from yellow to blue) in Fig. 6e, where the arrows point out the buckling events.

Summing the calculated energies required to bend each of the links, we estimated the minimum energy stored in the network as$H_{total}=\sum_{i=0}^{k}H_{i}=\sum_{i=0}^{k}\int_{0}^{L_{i}}\kappa_{i}ds/2R_{c_{i}}^{2}$, where *L* is the bundle length, *κ* is the bending rigidity, *k* is the number of buckled bundles in the network, *s* is the arc length and *R*_*c*_ is the radius of curvature of the buckled bundles. Fig. 6f shows a network before and after relaxation; the former color-coded for the stored energy and the latter shown in greyscale. The arrows indicate some typical cases of buckling of actin bundles. We obtained stored energies of 100–200 *k*_*B*_ *T*, indicating that a substantial amount of internal stress was generated during the network formation (Fig. 6g). It has been shown that the formation of actin networks via cross-linking proteins builds up an internal stress, resulting in kinetically trapped networks and dissipating stress through the transient unbinding events of the cross-linking molecules [28–30]. Here, we directly observed the stored stress in Mg^2+^-induced networks as it was released and utilized in the buckling of bundles during the de-bundling process. The de-bundling process was much slower (≈ 30 min) with bigger bundles dissociating into small bundles as well as directly into single filaments (Fig. 5b). Such release of tension was not seen during the disassembly of clusters formed by short filaments.

### Depletion interaction-induced emerging networks form spindle-like intermediate structures

Depletion interactions are non-specific, which tend to assemble large objects together in a crowded environment of sufficient number of small objects. These interactions are primarily entropic in origin as first described by Asakura and Oosawa [31]. The intracellular environment is a crowded one, where 20–30% of the volume is occupied by soluble proteins and other macromolecules [32]. We used PEG polymers (M. W. 8000) as the crowding agents to mimic the cytoplasmic soup in a cell.

**Fig 7:**
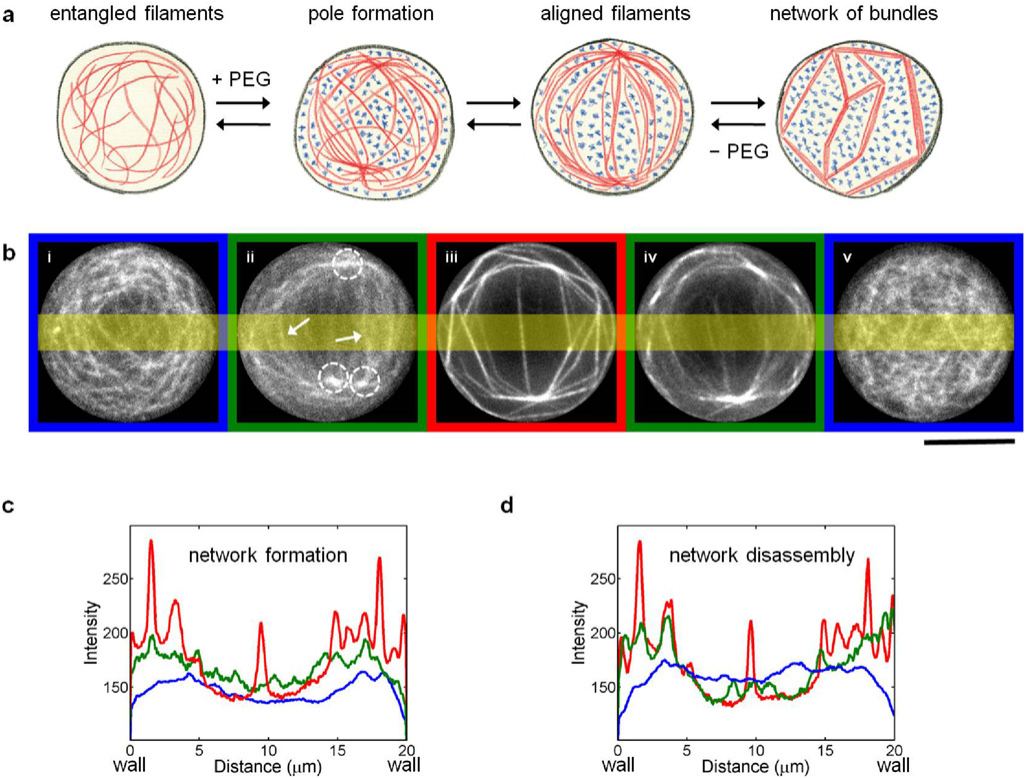
Spindle-like intermediate structure during the PEG-induced actin network formation. (a) Sketch of network formation by depletion interactions. (b) Time-lapse images showing the network formation induced by PEG polymers (5% w/v, M. W. 8000), and the subsequent disassembly after their depletion. The scale bar represents 10 μm. (c, d) Mean intensity distribution through the highlighted area showing the spatial distribution of fluctuating filaments (blue), aligned filaments (green) and bundles (red) during (c) network formation and (d) disassembly.

Addition of crowding agents to long actin filaments resulted in an interesting hierarchical assembly with intriguing steps, sketched in Fig. 7a and shown as time-lapse images in Fig. 7b. Upon addition of PEG, actin filaments clustered at certain positions (termed as poles) to produce an aster-like appearance. The poles, visualized as bright spots (indicated by the dotted circles in Fig. 7b-ii), were often situated close to the confinement boundary, diagonally opposite to each other. The number of poles varied between 2–3 and the average distance between the poles increased from about 7 μm for small confinements up to 14 μm for bigger confinements. Single filaments as well as few small bundles were shared between these poles (indicated by arrows in Fig. 7b-ii). The shared filaments eventually aligned along their long axes in between the poles, resulting in the reduction of their fluctuations. This structure had a remarkable similarity to the spindle apparatus, which is a structure primarily based on microtubules and microtubule motor proteins formed during the eukaryotic cell division and essential for chromosome segregation [33]. The poles could be compared to the centrosomes, while the stretched and aligned actin filaments could be compared to the kinetochore microtubules aligned between the centrosomes.

The ‘spindle’ stage lasted for about 8 min, followed by a bundling transition, in which the aligned filaments finally came together, forming straight and rigid bundles (Fig. 5e). Fig. 7c shows the intensity profiles across the microchambers perpendicular to the pole axis (highlighted area in Fig. 7b) at various stages, viz., fluctuating filaments (blue), aligned filaments (green) and bundles (red). The green profile exhibits small, but definite perturbations associated with the sets of aligned filaments. These perturbations further developed into sharp spikes, corresponding to bundles. Thus, the aligned filaments acted as preliminary structures that finally evolved into bundles.

In contrast to the disassembly of Mg^2+^-induced networks, the de-bundling process of PEG-induced networks virtually represented a reversal of the bundling process, wherein the transition from bundles back to aligned filaments was observed (Fig. 7d). Since PEG polymers were not present inside the bundles and did not have any direct interactions with F-actin, they were readily removed from the system. As the poles disintegrated, filaments lost their alignment and formed an entangled network. Cluster formation by short filaments showed no such spindle-like structure and the segregated clusters formed randomly.

### Filamin-induced emerging networks form ring-like irreversible structures

Numerous ABPs such as espin, fascin, α-actinin and filamin can induce actin bundling *in vivo* as well as *in vitro* [1]. Of these, we chose to induce actin bundling by filamin dimers (ratio of actin to filamin (*R*) = [filamin]/[actin] = 0.1). As the filamin dimers diffused into the microchambers, specific protein-protein interactions between filamin and F-actin led to the bundling of actin filaments. Requiring approximately one hour for completion, the bundling reaction with filamin was comparatively slow, due to the low diffusion coefficient (~ 40 μm^2^/s) and low concentration used (0.3 μM compared to 50 mM Mg^2+^ and 6.3 mM PEG), in addition to the highly specific interactions between F-actin and filamin.

The filamin-induced bundles exhibited a distinctly curved shape. The curved bundles ultimately met each other to form closed ring-like structures (Fig. 2c-iv), similar to those previously observed for filamin-induced bundle formation in vesicles [34] and in ring-like bundles formed during *Drosophila* oogenesis [35]. The circular walls of the confinements, however, were not responsible for the curved bundles, as the same scenario was obtained in square or triangular microchambers (S6 Fig.). Rather, the curved nature seemed to be a consequence of the intrinsic flexibility of filamin [36, 37] and bent conformations of long filaments within confinements. On the other hand, short and rigid actin filaments yielded straight bundles (Fig. 3c).

Surprisingly, the obtained networks could not be dissolved back to the entangled filaments (S6 Video and S12 Video). Even after 12 hours of continuous flow of filamin-free solution in the controlling channel, networks were clearly visible and intact. It has been reported that the extreme stability of bundles, induced by fascin and α-actinin, endures for over half an hour with continuous protein-free wash [38]. This makes it likely that filamin-induced bundles will be at least as stable since filamin has a higher binding affinity compared to fascin and α-actinin [37]. It is possible that *in vivo*, some other competitive proteins might aid in the de-bundling process. In fact, there are more than 20 proteins known to affect the F-actin binding affinity of filamin [1].

## Discussion

Being one of the major cytoskeletal proteins, elucidating actin dynamics is important in order to understand how the cell functions. The viscoelastic properties shown by actin-based structures have been abundantly investigated by various means including rheology and optical tweezers [7–9]. Even then, the involved microstructure dynamics that are responsible for the mechanical properties of actin networks have received less attention, especially regarding their disassembly [11]. This is mainly due to the fact that most experimental systems are closed and one needs an open system in order to study step-by-step reaction sequences. We have previously shown the basic functioning of diffusion-controlled micro-confinements using divalent cations as bundling agents [20]. In the same study, we also showed evaporation-assisted networks of actin bundles, whose properties strongly depended on the confinement geometry. Realizing the potential of our system, in this article we focused on the spatiotemporal evolution of networks and bundles in a step-by-step manner. Performing a real time analysis in an open system facilitated the elucidation of reversible actin bundling, which is not possible in closed systems. Using diffusion-controlled addition and depletion of bundling agents, we observed the hierarchical nature of bundling.

We used a minimal system comprised solely of actin filaments and specific bundling agents within micro-confinements and observed significant self-organization of F-actin in the form of emerging networks of actin bundles. This emergence could be attributed to the simultaneous sharing of filaments within multiple actin bundles (network links). When this sharing of filaments was eliminated by shortening the actin filaments down to 1 μm, the network formation was dramatically suppressed and isolated clusters of bundles formed instead. Thus, we found two distinct length regimes: short filaments, where an exclusive bundling process is observed, and long filaments, where bundling is accompanied by percolation to form a connected network. We used three distinct bundling mechanisms, viz., counterion condensation (Mg^2+^ ions), depletion interactions (PEG polymers) and specific protein-protein interactions by ABPs (filamin dimers), to induce F-actin bundling. Though each of these processes have distinct characteristics, they shared a common theme of hierarchy, where filaments first formed small bundles which later joined to form bigger bundles. The diffusion-controlled depletion of the bundling agents from the micro-confinements led to the network disassembly. The observed hierarchical assembly and disassembly processes depended on the bundling agents (the mechanism and the diffusion coefficient), the length of actin filaments and the confinement sizes. Using time-lapse epifluorescence microscopy and intensity-based image analysis, we tracked the evolution of filaments, small bundles and big bundles for each of the processes. Furthermore, we used simple first order kinetic models, which fit many of the observed step-by-step dynamics.

Typical sizes of eukaryotic cells range from ~ 10 μm for yeast cells up to ~ 50 μm for plant and animal cells [33]. We used a similar size range of the micro-confinements to perform the experiments, making them relevant on a biological length scale. The average actin filament length in cell extracts, along with the reconstituted cytoskeletal networks, is in the order of several hundred nanometers and the distribution of F-actin lengths can be quite broad, up to 13 μm [39, 40]. The actin concentration *in vivo* is more than an order of magnitude higher (70–150 μM) than that used in our experiments [40]. Hence, network formation is likely even for shorter filaments (< 1 μm) *in vivo*, and the observed network formation is highly plausible in cells. Since this percolation can be directly correlated to intracellular conditions, it is likely that many of the *in vivo* actin-based structures will have a long-range connectivity resulting in an enhanced responsiveness to internal and external signals. Also, most experiments and theoretical models tend to ignore the polyelectrolyte nature of F-actin and molecular crowding [11], which we elegantly addressed using a microfluidic set-up, thus elaborating on the feasible cytoskeletal dynamics.

Though the concentration of divalent cations such as Mg^2+^ is in the range of few mM *in vivo* [14], the total Mg^2+^ levels can be as high as 10 mM, where much of it is incorporated in various intracellular structures [41]. Thus, the contribution of counterion condensation-induced F-actin bundling is plausible in the cell, especially in a local environment (e.g. endoplasmic reticulum) where the divalent concentration may be sufficiently high. During network formation, we observed a zipping behavior where filaments joined together lengthwise to form bundles. The zipping velocity was up to 12 μm/s and the force generated during the zipping process was about 0.15 pN. The sharing of filaments between the bundles resulted in a stressed network. Depleting the bundling agents released this stress, allowing us to estimate the energy stored in the networks to be between 100 − 200 *k*_*B*_ *T*. This provided a direct evidence for internal stress generation in a network, without any active damage to it. Hence, we have demonstrated that counterion condensation is sufficient to generate internal stress within an actin network without any need of specific cross-linking proteins.

The interior of a cell is a highly crowded environment and under such conditions, bundling of actin filaments by depletion interactions becomes very relevant [32]. The crowding agents induced a self-assembled ‘spindle-like’ intermediate structure, where F-actin condensed at few regions near the confinement boundary with shared sets of aligned filaments stretched between them. This spindle-like structure then transformed into a network of bundles. Although the components of the eukaryotic spindle mainly involve microtubules and associated motor proteins [33], its similarity with the observed structure is indeed striking. The PEG-induced formation of spindle-like structure is entropically driven and emphasizes the presence of comprehensive (“global”) cellular processes. Also, there is a growing evidence that F-actin and myosin motors are possibly involved in spindle assembly and function [42].

The relevance of bundling and network formation via ABPs is obvious owing to their sheer number inside the cell, where each of the proteins are highly specific and are evolved to perform specific tasks. The filamin-induced networks consisted of curved, rather than straight bundles owing to the intrinsic flexibility of filamin [37, 38] and bent conformations of long filaments within confinements. This gave rise to ring-like structures with many bifurcations. We could not disassemble the obtained networks back to entangled filaments, even after 12 hours of filamin-free environment, probably due to the highly specific interactions between F-actin and filamin.

Taking into account the dynamic nature of cells and the intracellular actin bundle-based structures [1], our *in vitro* experiments of step-by-step assembly and disassembly of actin bundles shed more light on the bundling, and particularly, the de-bundling processes. Also, it is probable that *in vivo*, these distinct mechanisms work together in a consortium. Finally, our study demonstrates that a controlled manipulation of dynamic minimal systems, where one can reversibly control reactions in a sequential manner, can unveil novel structures and properties of macromolecules, leading to a better understanding of the complex biological world.

## Materials and Methods

### Soft lithography, microfluidic device preparation and pre-treatment

We used standard soft lithography techniques to fabricate microfluidic devices. SU8 negative photoresists (Microchem, Newton, MA, USA) were spin coated on clean silicon wafers (Si-Mat, Kaufering, Germany) and exposed to ultraviolet light through appropriate chromium masks (ML&C GmbH, Jena, Germany) to obtain masters. To produce multi-height devices, i.e., a device with different heights for different substructures, multi-layer photolithography was performed using a MJB4 mask aligner (SUSS MicroTec AG, Garching, Germany). In order to produce a master with 0.5 μm thick microchambers and a 5.5 μm high controlling channel, first layer was of SU8 2000.5 (MicroChem, Newton, USA) and the second one was of SU8 3005 (MicroChem, Newton, USA). Polydimethylsiloxane (PDMS) and cross-linker (Sylgard 184, Dow Corning GmbH, Wiesbaden, Germany) were mixed in the mass ratio 10:1, degassed and poured on the masters followed by baking at 80°C for at least 4 hours. Cured PDMS was peeled off from the wafer, punched (to insert the tubings later on), cleaned with isopropanol, dried with nitrogen and then covalently bonded to a clean glass slide after a plasma treatment at 2 mbar for 30–40 s in a plasma cleaner (Harrick Plasma, NY, USA). To minimize the interactions of reaction components with microchamber walls, the freshly prepared device was rinsed with 1 mg/mL PLL(20)-g[3.5]-PEG(2) (SuSoS AG, Dübendorf, Switzerland) for about 30 min and then with water. The device was constantly equilibrated with water in order to eliminate evaporation through PDMS.

### Proteins and buffers

Actin from rabbit skeletal muscle, fluorescent Atto488-Actin from rabbit skeletal muscle, filamin from turkey smooth muscle and cytoplasmic gelsolin from porcine smooth muscle were purchased in the form of lyophilized powders from Hypermol EK, Bielefeld, Germany. Polymerization buffer (1 M KCl, 0.1 M imidazole (pH 7.4), 10 mM ATP, 20 mM MgCl_2_) and dilution buffer (2 mM Tris-HCl (pH 8.2), 0.4 mM ATP, 0.1 mM CaCl_2_, 0.5 mM DTT (dithiotreitol)) were also purchased in the form of lyophilized powders from Hypermol EK, Bielefeld, Germany. The powders were reconstituted in Millipore water to the required concentrations and kept on ice or in small aliquots at -80°C. To induce actin polymerization, polymerization buffer was added to the actin solution in 1:9 ratio. Final composition of the actin solution was 3 μM actin, 1.4–1.5 mM ATP, 100 mM KCl, 2 mM MgCl_2_, 0.1–0.2 mM CaCl_2_, 0.5–0.6 mM DTT, 10 mM imidazole, 2–2.4 mM Tris-Cl (pH 7.4) and 0.1–0.2% disaccharides. Actin:Atto488-actin ratio was 10:1. In case of experiments involving gelsolin, CaCl_2_ concentration was increased to 0.2 mM.

### Experimental procedure

To initiate the experiment, a semi-dilute (3 μM) actin solution, along with polymerization buffer, was injected in a pre-treated device using appropriate tubing and syringe pumps (Cetoni GmbH, Germany). The polymerizing filaments became confined within the microchambers due to their lowered diffusion coefficient, compared to monomers and due to an additional hindrance presented during the transit through the narrow connecting channels. Eventually, a steady-state of entangled fluctuating actin filaments was reached in a few hours. To initiate the bundling process, a solution with the same actin composition plus the required concentration of the bundling agents was introduced into the controlling channel. As the bundling agents entered the microchambers by diffusion, the bundling process started and was recorded by time-lapse microscopy. To start the de-bundling process, the solution in the controlling channel was changed back to the original solution, i.e., actin solution without any bundling agent.

### Fluorescence imaging

An Olympus IX81 inverted microscope equipped with a fluorescence illumination (X-Cite Series 120 Q), an appropriate filter set and a 100x (N.A. 1.49) UApo N oil immersion objective (Olympus, Tokyo, Japan) was used to perform experiments. The images were recorded with a pco.edge camera (PCO AG, Kelheim, Germany) using pco.camware software (0.1–20 frames per second, 20–50 ms exposure times depending on the type of the experiment).

### Image processing and analyses

Images were processed using ImageJ (1.47k, Wayne Rasband, National Institute of Health, USA) and MATLAB (R2009a, R2012a, The MathWorks Inc.). To analyze network dynamics, tiff stacks were background subtracted, any possible artifacts were removed and then analyzed with MATLAB using self-written scripts.

## Supporting Information

S1 Fig Temporal evolution of concentration distributions of actin monomers and bundling agents within the microchambers. Concentration distributions over time of (a) actin monomers, (b) Mg^2+^ ions, (c) PEG polymers and (d) filamin dimers, with a fixed concentration at distance *x* = 0 (corresponding to the start of the connecting channel) and a fix, no-flux boundary at *x*_*max*_ = 50 μm (denoting the farthermost microchamber boundary from the main channel). The uppermost (magenta) profiles in each of the plots correspond to the time *t*_0.95_, when *C*(*x*_*max*_,*t*_0.95_) ≈ 0.95*C*_0_. See S1 Text for more information.
(EPS)

S2 Fig Actin bundle rings in small confinements. Representative examples of rings of actin bundles induced by Mg^2+^ ions, PEG polymers and filamin dimers in the smallest confinements. Scale bar represents 5 μm.
(EPS)

S3 Fig Validation of the linear relationship between fluorescence intensity of a bundle and the number of filaments it contains. Representative examples showing intensity profiles of two bundles (blue and green) and the intensity profile of the bigger bundle they form (red). Note that in each case, sum of the fluorescence intensities of the two smaller bundles matches up with the intensity of the bigger bundle. Bundling is induced by (a) Mg^2+^ ions, (b) PEG polymers and (c) filamin dimers.
(EPS)

S4 Fig Frequency histograms of the estimated number of filaments inside the bundles. Frequency histograms of the greyscale values representing bundles induced by (a, b) Mg^2+^ ions, (c, d) PEG polymers and (e, f) filamin dimers. (a), (c) and (e) refer to bundles of long filaments (≥ 10 μm) while (b), (d) and (f) refer to bundles of short filaments (1 μm). The solid line is a polynomial fit for each histogram as a guide to locate the peaks denoted by arrows. The estimated number of filaments per bundle is also shown in each graph (lower *x*-axes). Bundles within networks show discernible peaks (indicated by arrows), while clusters do not show any dominant peaks but more of a decaying or a homogenous profile. The bundles are divided into two categories: small bundles (*b*) and big bundles (*bb*). The regions chosen for these two categories for each of the six histograms are shown. These regions are selected based on the individual nature of the histograms, this categorization of bundles into b and *bb* is performed in order to convey the hierarchical nature of the bundling process.
(EPS)

S5 Fig Distribution of small and big bundles within networks formed by long filaments and clusters formed by short filaments. The small and big bundles are defined as per S3 Fig. The scale bar represents 10 μm.
(EPS)

S6 Fig Filamin-induced curved actin bundles in square and triangular confinements. Representative examples of curved actin bundles induced by filamin dimers in square as well as triangular confinements. Scale bar represents 10 μm.
(EPS)

S1 Text Temporal evolution of concentration distributions of actin monomers and bundling agents within the microchambers. (DOCX)

S2 Text Kinetic models used to fit the observed hierarchical assembly and disassembly reactions. (DOCX)

S1 Video Network formation, induced by Mg^2+^ ions (50 mM), 3x real time. (AVI)

S2 Video Network formation, induced by PEG polymers (5% w/v), 333x real time. (AVI)

S3 Video Network formation, induced by filamin dimers (*R* = 0.1), 333x real time. (AVI)

S4 Video Network (Mg^2+^-induced) disassembly, 141x real time. (AVI)

S5 Video Network (PEG-induced) disassembly, 333x real time. (AVI)

S6 Video Network (filamin-induced) disassembly, 1800x real time. (AVI)

S7 Video Cluster formation, induced by Mg^2+^ ions, 6x real time. (AVI)

S8 Video Cluster formation, induced by PEG polymers (5% w/v), 333x real time. (AVI)

S9 Video Cluster formation, induced by filamin dimers (*R* = 0.1), 333x real time. (AVI)

S10 Video Cluster (Mg^2+^-induced) disassembly, 180x real time. (AVI)

S11 Video Cluster (PEG-induced) disassembly, 10x real time. (AVI)

S12 Video Cluster (filamin-induced) disassembly, 600x real time. (AVI)
