# Supplementary material for: Real-Time Dynamics of Emerging Actin Networks in Cell-Mimicking Compartments
Source: PLoS One. 2015 Mar 18;10(3):e0116521. doi: 10.1371/journal.pone.0116521 (PMC4364982; doi:10.1371/journal.pone.0116521)
Supplement: S1 Text — (DOCX) [file pone.0116521.s007.docx]

**Temporal evolution of concentration distributions of actin monomers and bundling agents within the microchambers.**

We looked at how the concentration distribution evolves with time for a particular molecular species along a line (one dimension). In our experiments, the concentration at distance $x$ = 0, which corresponds to the start of the connecting channel, never changes and always remains $C_{0}$, since the solution in the controlling channel is constantly getting renewed. We also have a fixed, no-flux boundary condition, the microchamber wall, so that after a sufficient period of time, $C\left(x,\infty\right)=C_{0}$. The equation for concentration distribution along a line with fixed concentration $C_{0}$ at $x$ = 0 and a fixed, no-flux boundary at the end $x= x_{max}$, is given as

$$C\left(x,t\right)=C_{0 }\left(1+\text{erf}\left(\frac{2x_{max}}{\sqrt{4Dt}}\right)-\text{erf}\left(\frac{x}{\sqrt{4Dt}}\right)-\text{erf}\left(\frac{2x_{max}- x}{\sqrt{4Dt}}\right)\right)$$

Figure S1 shows how the concentration distributions for G-actin and different bundling agents evolve and finally converge with time. The used diffusion coefficients are $D_{actin}$ = 91 μm^2^/s, $D_{Mg}$ = 2000 μm^2^/s, $D_{PEG}$ = 46 μm^2^/s and $D_{filamin}$ = 39 μm^2^/s.
