# Supplementary material for: Real-Time Dynamics of Emerging Actin Networks in Cell-Mimicking Compartments
Source: PLoS One. 2015 Mar 18;10(3):e0116521. doi: 10.1371/journal.pone.0116521 (PMC4364982; doi:10.1371/journal.pone.0116521)
Supplement: S2 Text — (DOCX) [file pone.0116521.s008.docx]

**Kinetic models used to fit the observed hierarchical assembly and disassembly reactions.**

All the bundling and de-bundling reactions were approximated as first order reaction sequences. By integrating the differential equations, the time-dependent concentration of reaction components was found out.

Assembly of Mg^2+^-induced networks and clusters; assembly of filamin-induced networks:

In simplest terms, the bundling process could be expressed as

|  | $f\to^{k_{1}} b\to^{k_{2}} bb,$ | (1) |
| --- | --- | --- |

where $f$ denotes filaments, $b$ denotes small bundles and $bb$ denotes big bundles. The differential equations were expressed as follows:

|  | $\frac{d\left[f\right]}{dt}=-k_{1}\left[f\right],$ | (2) |
| --- | --- | --- |
|  | $\frac{d\left[b\right]}{dt}=k_{1}\left[f\right]-k_{2}\left[b\right],$ | (3) |
|  | $\frac{d\left[bb\right]}{dt}=k_{2}\left[b\right].$ | (4) |

Integrating equation 2, substituting the solution in equation 3 and following the mass balance led to the following solutions for the time-dependent concentrations of $f$, $b$ and $bb$.

|  | $\left[f_{t}\right]=\left[f_{0}\right]e^{-k_{1}t},$ | (5) |
| --- | --- | --- |
|  | $\left[b_{t}\right]=\frac{k_{1}}{k_{2}-k_{1}}\left[f_{0}\right]\left(e^{-k_{1}t}-e^{-k_{2}t}\right),$ | (6) |
|  | $\left[bb_{t}\right]=1-\left[f_{t}\right]-\left[b_{t}\right].$ | (7) |

The dashed lines in Figure 5b and 5c are the fits obtained using Equation 5 and Equation 7 for the evolution of filaments and big bundles, respectively. Using the obtained rate constants, we were satisfactorily able to reproduce the evolution of small bundles (solid lines).

Disassembly of Mg^2+^-induced networks and clusters:

A simplistic reaction sequence of the de-bundling process could be written as

|  | $bb\to^{k_{3}} b\to^{k_{4}} f,$ | (8) |
| --- | --- | --- |
|  | $bb\to^{k_{5}} f.$ | (9) |

The additional reaction (equation 9) where bigger bundles directly dissociated into filaments was neglected because the fraction of $bb$ was low enough to ignore their conversion to filaments, thus simplifying the model. The differential equations were expressed as follows:

|  | $\frac{d\left[bb\right]}{dt}=-k_{3}\left[bb\right],$ | (10) |
| --- | --- | --- |
|  | $\frac{d\left[b\right]}{dt}=k_{3}\left[bb\right]-k_{4}\left[b\right],$ | (11) |
|  | $\frac{d\left[f\right]}{dt}=k_{4}\left[b\right].$ | (12) |

Integrating equation 10, substituting the solution in equation 11 and following the mass balance led to the following solutions for the time-dependent concentrations of $f$, $b$ and $bb$.

|  | $\left[bb_{t}\right]=\left[bb_{0}\right]e^{-k_{3}t},$ | (13) |
| --- | --- | --- |
|  | $\left[b_{t}\right]=\frac{\left(\left[b_{0}\right]k_{3}-\left[b_{0}\right]k_{4}+\left[bb_{0}\right]k_{3}\right)e^{-k_{4}t}}{k_{3}-k_{4}}-\frac{\left[bb_{0}\right]k_{3}e^{-k_{3}t}}{k_{3}-k_{4}},$ | (14) |
|  | $\left[f_{t}\right]=\left[bb_{0}\right]+\left[b_{0}\right]-\left[bb_{t}\right]-\left[b_{t}\right].$ | (15) |

The dashed lines in Figure 5d are the fits obtained using Equation 13 and Equation 15 for the evolution of big bundles and filaments, respectively. Using the obtained rate constants, we were able to replicate the evolution of small bundles (solid lines).

Disassembly of PEG-induced networks:

The overall disassembly process was written as

|  | $bt\to^{k_{3}} p\to^{k_{4}} f,$ | (16) |
| --- | --- | --- |
|  | $bb\to^{k_{5}} f,$ | (17) |

where $bt$ denotes small as well as big bundles since they decayed at the same rate and $p$ denotes spindle-like structure (mainly the poles in addition to the aligned filaments) . Equation 17 was neglected for the same reason as neglecting Equation 9. The involved differential equations were as follows:

|  | $\frac{d\left[bt\right]}{dt}=-k_{3}\left[bt\right],$ | (18) |
| --- | --- | --- |
|  | $\frac{d\left[p\right]}{dt}=k_{3}\left[bt\right]-k_{4}\left[p\right],$ | (19) |
|  | $\frac{d\left[f\right]}{dt}=k_{4}\left[p\right].$ | (20) |

Integration of equation 18, substituting the solution in equation 19 and following the mass balance resulted in the solutions for the time-dependent concentrations of $f$, $p$ and $bt$.

|  | $\left[bt_{t}\right]=\left[bt_{0}\right]e^{-k_{3}t},$ | (21) |
| --- | --- | --- |
|  | $\left[p_{t}\right]=\frac{k_{3}}{k_{4}-k_{3}}\left[bt_{0}\right]\left(e^{-k_{3}t}-e^{-k_{4}t}\right),$ | (22) |
|  | $\left[f_{t}\right]=1-\left[p_{t}\right]-\left[bt_{t}\right].$ | (23) |

The dashed lines in Figure 5f are the fits obtained using Equation 21 and Equation 23 for the evolution of bundles and filaments, respectively. Using the obtained rate constants, we were able to replicate the evolution of spindle-like structure (solid line).

Assembly of PEG-induced cluster formation and filamin-induced cluster formation:

For these processes, small bundles and big bundles evolved almost at the same rate suggesting that two parallel reactions were taking place which could be expressed as

|  | $f\to^{k_{1}} b,$ | (24) |
| --- | --- | --- |
|  | $f\to^{k_{2}} bb.$ | (25) |

The differential equations were expressed as follows:

|  | $\frac{d\left[f\right]}{dt}=-(k_{1}+k_{2})\left[f\right],$ | (26) |
| --- | --- | --- |
|  | $\frac{d\left[b\right]}{dt}=k_{1}\left[f\right],$ | (27) |
|  | $\frac{d\left[bb\right]}{dt}=k_{2}\left[f\right].$ | (28) |

Integrating equation 26 and substituting the solution in equation 27 and equation 28, gave the time-dependent concentrations $f$, $b$ and $bb$.

|  | $\left[f_{t}\right]=\left[f_{0}\right]e^{\left(-(k_{1}+k_{2}\right)t)},$ | (29) |
| --- | --- | --- |
|  | $\left[b_{t}\right]=\frac{k_{1}}{k_{1}+k_{2}}\left[f_{0}\right]\left(1-e^{\left(-(k_{1}+k_{2}\right)t)}\right),$ | (30) |
|  | $\left[bb_{t}\right]=\frac{k_{2}}{k_{1}+k_{2}}\left[f_{0}\right]\left(1-e^{\left(-(k_{1}+k_{2}\right)t)}\right).$ | (31) |

The dashed lines in Figure 5b and Figure 5e are the fits obtained using Equation 29 and Equation 31 for the evolution of filaments and bundles, respectively. Using the obtained rate constants, we could trace the evolution of small bundles (solid lines).

We were not able to obtain simple kinetic models for assembly of PEG-induced networks and disassembly of PEG-induced clusters, as the observed evolution of components was not possible to express in terms of first order sequence approximation. An exponential decay (Equation 5) was used to fit the evolution of filaments in case of PEG-induced networks to obtain $k_{1}$.
